# Low-Temperature Sintering of a New Bioactive Glass Enriched with Magnesium Oxide and Strontium Oxide

**DOI:** 10.3390/ma15186263

**Published:** 2022-09-09

**Authors:** Devis Bellucci, Valeria Cannillo

**Affiliations:** Dipartimento di Ingegneria “Enzo Ferrari”, Università degli Studi di Modena e Reggio Emilia, Via P. Vivarelli 10, 41125 Modena, Italy

**Keywords:** bioactive glass, sintering, crystallization, bioactivity, therapeutic ions

## Abstract

The recent research on bioactive glasses (BGs) has mainly moved on two fronts: (1) introducing ions of therapeutic interest in their composition and (2) the development of scaffolds, fibers, coatings and sintered products starting from BGs in powder form. In this case, the main obstacle to overcome is that BGs rapidly crystallize during heat treatments, thus transforming into glass-ceramics with low reactivity, slow ion release and, eventually, poor mechanical properties. Here an innovative bioactive glass (BGMS_LS), capable of responding to the main limitations of commercial BGs, is presented. The new material contains strontium and magnesium, whose therapeutic relevance is well known, and can be sintered at extraordinarily low temperatures without crystallizing, thus keeping all of its biological potential intact.

## 1. Introduction

At the end of the 1970s, the discovery of bioactivity marked a turning point in the research on biomaterials: in fact, for the first time, a synthetic material was able to bind to the human tissues. Since then, 45S5 Bioglass^®^ (45S5), the first bioactive glass developed and progenitor of glasses for biomedical use, has been widely used in orthopedics and dentistry [1]. In more recent years, it has been demonstrated that the ions released from 45S5 during its dissolution may influence the behavior of cells, thus favoring specific cellular events such as proliferation, differentiation, angiogenesis and bone metabolism [2]. This fact has paved the way for the use of BGs in the field of tissue regeneration, stimulating the investigation on the biological effects of the ions they release.

Unfortunately, BGs have inherited a number of disadvantages from common glasses, such as brittleness and, in particular, the tendency to crystallize during the heat treatments which are necessary to sinter powders or to deposit bioactive coatings on metal substrates. The crystallization is reported to inhibit the glasses’ bioactivity and to greatly slow down their ionic release, thus limiting the biological responsiveness of these innovative materials; for example, the famous 45S5 can be sintered only at very high temperatures, of the order of 1000 °C, thus producing a widely crystallized material which is potentially unstable once implanted in the body, as the glass phase is degraded more rapidly than the crystalline phase [3,4]. This obvious disadvantage is due to the 45S5 atomic set-up which, at the same time, gives the glass its marked bioactivity, as well as the ability to degrade in contact with biological fluids and to form a surface layer of hydroxyapatite. In fact, the ability of BGs to bond to bone is mediated through the precipitation of an interfacial bone-like hydroxyapatite film when BGs are placed in contact with physiological fluids in vivo [5]. Compared to common silicate glasses, 45S5 and, more generally, BGs, contain large amounts of modifier oxides, in particular sodium oxide, which disrupt the silicate network; this favors the glass dissolution but, on the other hand, reduces the glass’ viscosity and increases the glass’ structural mobility, thus promoting crystallization of the glass.

Several attempts have been made to modify the 45S5 composition, for example, by replacing part (or all) of its calcium content with magnesium or zinc [6]; despite the fact that the obtained glasses were characterized by a decrease in crystallization tendency, the effect of such ions on the glass’ reactivity has not been completely clarified yet. On the other hand, the presence of calcium in the glass composition is important, both for its biological effects and because it contributes to the formation of apatite, so it does not seem appropriate to completely eliminate such an element from the glass composition.

Recently, a new class of BGs with a high crystallization temperature and marked biological response has been developed [7,8]. The pronounced biological activity of these materials was ascribable to the presence, in their composition, of both magnesium and strontium, whose therapeutic relevance has been confirmed by several studies [9,10]. In this work the glass composition has been further improved, increasing its sodium content and decreasing that of silicon with respect to [7], thus obtaining an even more reactive glass, named BGMS_LS. Moreover, thanks to its extraordinary low crystallization with respect to common BGs, BGMS_LS has a very low sintering temperature (about 686 °C), among the lowest ever reported in the literature. This implies not only obvious advantages from an economic point of view, but also the possibility of sintering the new BGMS_LS while keeping it completely amorphous; in this way, the final product will preserve its biological activity, in vivo dissolution rate and ionic release. Therefore, BGMS_LS is very promising, in particular for the fabrication of products which requires a thermal treatment, such as scaffolds for bone tissue regeneration.

## 2. Materials and Methods

BGMS_LS (composition, in mol%: 7.1 Na_2_O; 31.3 CaO; 5.0 MgO; 10.0 SrO; 2.6 P_2_O_5_; 44.0 SiO_2_) was produced by melt-quenching [11]. The glass powders were used for differential thermal analysis (DTA, Netzsch Differential Thermal Analyzer STA 429 CD, Netzsch-Gerätebau GmbH, Selb, Germany) and heating microscopy (HM, Misura 3.32; Expert System Solutions, Modena, Italy) to measure the characteristic temperatures of the material. Glass powders were pressed in disk form and thermally treated at 686 °C for 3 h. The sintering attitude of BGMS_LS was estimated by the sintering parameter *S_c_* = T_c_onset_ − T_s_ [12] and by evaluating the volume shrinkage Δ_%_ of the glass disks after the thermal treatment. The following equation

$$\Delta_{\%}=\frac{d_{0}-d_{s}}{d_{0}}\cdot 100$$

was used to calculate Δ_%_, where *d*_0_ and *d_s_* are the nominal diameter of the press (load) piston and the measured diameter of the glass disks after sintering, respectively. The crystallization in sintered samples was excluded through X-ray diffraction (XRD, Philips PW3710, Almelo, The Netherlands); to this aim, the samples, crushed in powder, were scanned between 2θ = 10° and 2θ = 70° with steps of 0.02° and 6 s each step. Hardness and Young’s modulus of the sintered samples were measured with a micro-indentation technique by means of open platform equipment (CSM Instruments, Peseux, Switzerland) according to [13]. The in vitro bioactivity of the BGMS_LS disks was studied by immersing the samples for 7 and 14 days in a simulated body fluid solution (SBF), which has a similar ion concentration to that of human plasma [11,14]. Both the surface and cross section of the samples after and before immersion in SBF were observed in a SEM (Quanta 2000, FEI Co., Eindhoven, The Netherlands); the possible precipitation of apatite was studied by means of X-ray energy dispersion spectroscopy (EDS, Inca, Oxford Instruments, Abingdon, UK) and micro-Raman spectroscopy. To this aim, a Raman microscope spectrometer (Horiba Jobin-Yvon, Villeneuve D’Aseq, France) with a 632.8 nm-wavelength laser was employed. For each sample, 15 acquisitions of 30 s each were performed.

## 3. Results

**Figure 1:**
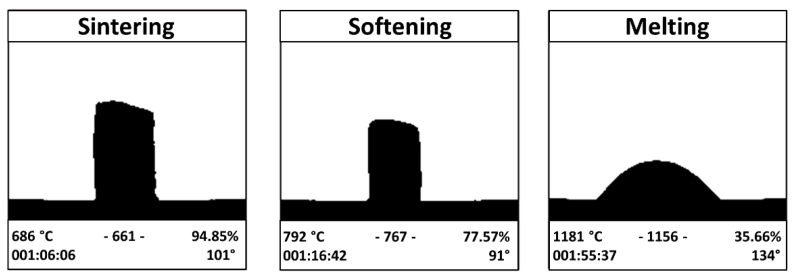
HM analysis of the BGMS_LS pressed powders.

The results of the HM analysis are reported in Figure 1; the characteristic temperatures of BGMS_LS, obtained from HM and DTA curve (not shown for the sake of brevity), are reported in Table 1, together with the glass’ mechanical properties, its *Sc* parameter and volume shrinkage Δ_%_.

**Figure 2:**
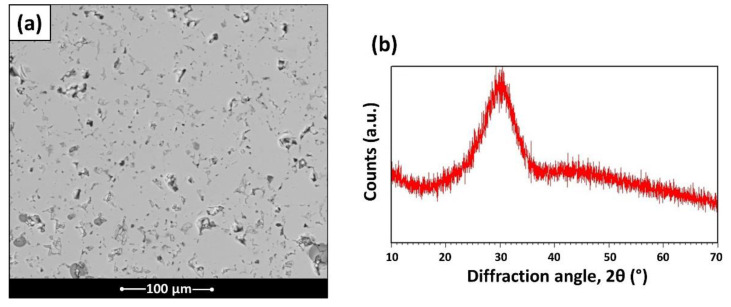
Sintered BGMS_LS: (**a**) cross section and (**b**) XRD diffractogram.

BGMS_LS starts to devitrify at 810 °C and the crystallization peak is reached shortly thereafter, at the extraordinarily high temperature of about 860 °C. Such a crystallization temperature is among the highest reported in the literature for BGs and represents an undoubted advantage with respect to 45S5, which starts to crystallize at temperatures of about 650 °C [6,15]. For this reason, despite the fact that 45S5 is well known for its reactivity, it suffers a narrow processing window (the temperature range between T_g_ and T_c_onset_, about 118 °C for 45S5 [6]) and poor sintering behavior, as the viscous flow is inhibited by the concomitant devitrification, which occurs close to glass transition. The novel glass here discussed is instead able to combine an excellent bioactivity with a high crystallization temperature, which allows the glass to be sintered at a remarkably low temperature (about 686 °C); this makes BGMS_LS one of the bioactive glasses with the lowest sintering temperature. Moreover, the processing window of BGMS_LS (~200 °C) is larger than that of 45S5 [6] and its sintering parameter *S_c_* is positive and high, which means that the kinetics of sintering and crystallization are independent and the sintering occurs prior to devitrification [8,12]. Such findings are confirmed by (1) the relatively high-volume shrinkage of the glass (see Table 1), whose sintering attitude is analogous to that of previously reported BGs with low tendency to crystallize [11], and (2) by the investigation of the cross sections of sintered BGMS_LS (Figure 2a), which shows an adequate densification (with the exception of some residual porosity). In addition, the XRD diffractograms acquired on the same samples (Figure 2b) are characterized by the classical trend of an amorphous glass, thus excluding a possible crystallization during the sintering process. Although the presence of micro-pores influences the mechanical performance of sintered ceramics, the hardness and the Young’s modulus (Table 1) of BGMS_LS are similar to those reported in the literature for sintered BGs [11].

**Figure 3:**
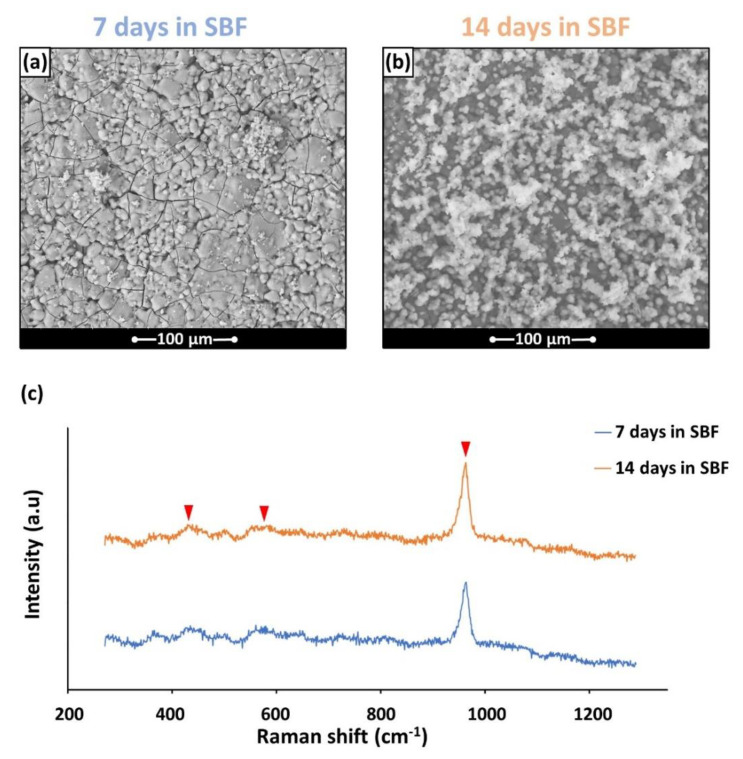
(**a**,**b**) The HA formation on BGMS_LS samples after soaking in SBF; (**c**) typical Raman spectra acquired on the HA precipitates. The arrows indicate the main peaks of HA.

**Figure 4:**
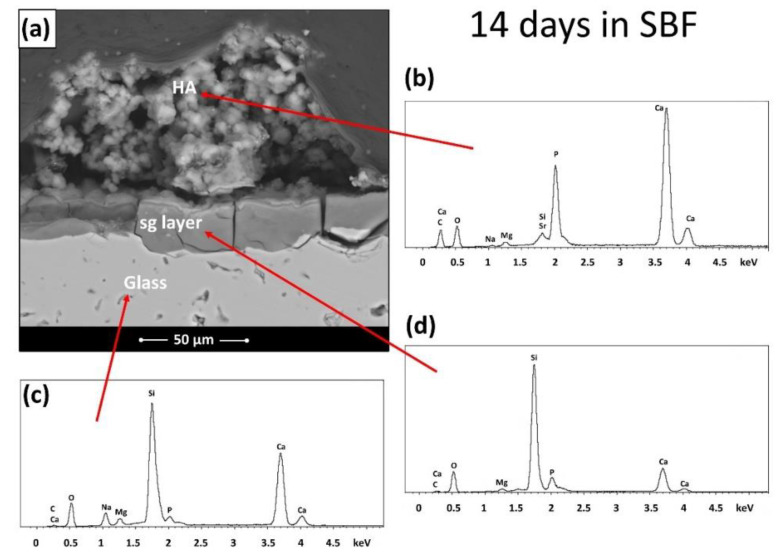
BGMS_LS samples after immersion in SBF for 14 days: (**a**) cross section and (**b**–**d**) EDS spectra.

The strong bioactivity of BGMS_LS was confirmed by means of an in vitro test in SBF, where it was possible to observe both the significance precipitation of apatite (see the white globular precipitates on the samples’ surface in Figure 3 and Figure 4) and the formation of a silica gel (sg) layer (see the cross-section shown in Figure 4 and the corresponding results of the EDS analysis), which precedes and accompanies the precipitation of the apatite itself [16]. The presence of apatite has been confirmed by the EDS results (Figure 4b), which report that the Ca/P ratio in the precipitates gradually approaches that of apatite [17,18], apart from local fluctuations. Moreover, the nature of the chemical species which formed on the samples during SBF tests was investigated by means of Raman spectroscopy. In fact, such technique is very useful in order to confirm the precipitation of apatite in vitro, as the Raman peaks related to the P–O vibration modes are particularly intense. In the spectrum of Figure 3c, acquired on the phosphorus and calcium rich precipitates after 7 and 14 days in SBF, it is possible to identify the main peaks ascribable to apatite, which arise from phosphate ions [19,20]. In more detail, the spectrum is dominated by a particularly sharp peak at ~960 cm^−1^, which, in the literature, is referred to as the ν_1_ symmetric stretching vibration of phosphate anions; moreover, it is possible to observe two further broad peaks which progressively emerge from the background, i.e., at ~430 cm^−1^ and 590 cm^−1^ (see the arrows in Figure 3c), where the literature reports the presence, for apatite, of peaks ascribable to the ν_2_ and ν_4_ bending modes of the (PO_4_)^3−^ group [19,20,21].

In summary, the marked bioactivity of BGMS_LS and its low-temperature sintering are ascribable to several competing factors, which derive from the composition of the glass itself: (1) the lower content of SiO_2_ compared to 45S5, which results in a weaker glass network, thus favoring the reactivity of the glass; (2) the lower content of Na_2_O, which is expected to promote its thermal stability; (3) the beneficial effect of MgO, which should improve the glass sintering by virtue of its higher field strength compared to calcium, although the debate on the role of magnesium in silicate glasses is still open [6]; (4) the presence of strontium, which should favor the viscous flow [11]; and (5) the increased entropy of mixing in the new multi-component BGMS_LS, which tends to favor the persistence of an amorphous disordered state, thus hindering crystallization. For these reasons, the marked reduction in alkaline oxides, combined with the addition of magnesium and strontium, whose biological relevance is ascertained, would seem to be an intriguing way to overcome the well-known limitations of common bioactive glasses.

## 4. Conclusions

Thanks to its composition, the new BGMS_LS exhibits exceptional thermal stability, which makes the glass sinterable at a much lower temperature than that of the “gold” standard 45S5. Despite the heat treatment, the sintered glass remains completely amorphous, making it possible to produce highly bioactive scaffolds and coatings. In view of a clinical use of BGMS_LS, the biological responsiveness of strontium and magnesium could make the new glass even more promising.

## Figures and Tables

**Table 1:** BGMS_LS: characteristic temperatures (T_g_—glass transition; T_s_—sintering; T_c_onset_—onset crystallization; T_c_—peak crystallization; T_m_—melting), sinterability parameter *S_c_*, volume shrinkage Δ_%_, Young’s modulus and hardness.

T_g_ (°C)	T_c_onset_ (°C)	T_c_ (°C)	T_s_ (°C)	T_m_ (°C)	Processing Window (°C)	*S_c_* (°C)	Shrinkage (%)	Young’s Modulus (GPa)	Hardness (Vickers)
610	810	860	686	1181	200	124	13.65 ± 0.56	69 ± 6	411.6 ± 88.5
